# Clinical Cases*Read before Central N. Y. Hom. Society, at Syracuse, March, 1882.

**Published:** 1882-04

**Authors:** C. Lippe

**Affiliations:** New York


### CLINICAL CASES.*

C. Lippe, M. D., New York.

Case I.—A lady had for several days frequent diarrhœic stools, commencing in the morning on rising, sudden urging, gushing, accompanied with flatulence, *the stool spattering all over the vessel;* the frequent movements had produced considerable prostration. A single dose of *Natrum sulphuricum* was given in the CM potency of Fincke. The frequent movements gradually ceased during the day, and no more medicine was given. The following morning the loose evacuations did not return, and the patient, with the exception of a little feeling of weakness, was well. *Sulphur* and *Aloes* have the morning diarrhœa, but the patient is compelled to arise from the desire to evacuate. The *Sulphur* patient has colic before a loose evacuation ; *Aloes,* rumbling of flatulence before stool.

Case II.—The patient complains of sore throat ; on examination, find ulcer on both tonsils more on the right side, with external swelling of that side of the throat ; painful to touch ; of course there is considerable inflammation and difficulty of swallowing. The history of the case informed me that the pain commenced on the left side and progressed to the other side. On experiment, found that cold liquids were easier swallowed than warm fluids. *Lachesis* CM (Fincke), was given one dose; the following day the patient reports *no better,* but is *no worse;* no medicine; the following day much

* Read before Central N. Y. Hom. Society, at Syracuse, March, 1882.

better; in a day was well. This was a clear case for the remedy, and the indications have been verified many times during the winter, especially the easier swallowing of cold fluids. *Lycopodium* has the swelling, pain and ulceration commencing on the right side, extending to the left, and the greater facility of swallowing warm liquids.

In these acute cases, when the proper remedy has been selected, and just enough, sufficient for that patient has been administered, it is often found that in the first twenty-four hours there is no improvement. If an improper (non-homœopathic) remedy had been administered, the case would progress from bad to worse; if too much medicine, or in too often-repeated doses, there would likely be an aggravation; but finding the case apparently in *statu quo*, my judgment was that the proper remedy had been given, and just enough, and the next day proved this view correct. In giving one dose and watching the case, experience has taught me that my cases convalesce more rapidly. In case of a progress of the disease after being twenty-four hours stationary, the symptoms being similar to the previous condition, then a powder of a different (generally higher) potency might be dissolved in water, a tea-spoonful given every one or two hours, for four or five hours, and the result waited for; if an improvement follows, no matter how slight, let the remedy act until its action has been exhausted.

